# Sulfation and Its Effect on the Bioactivity of Magnolol, the Main Active Ingredient of *Magnolia Officinalis*

**DOI:** 10.3390/metabo12090870

**Published:** 2022-09-15

**Authors:** Cong Xie, Wanyu Hu, Lili Gan, Bingxuan Fu, Xiaojie Zhao, Dafu Tang, Rongxin Liao, Ling Ye

**Affiliations:** 1 Clinical Pharmacy Center, Nanfang Hospital, Southern Medical University, Guangzhou 510515, China; 2 NMPA Key Laboratory for Research and Evaluation of Drug Metabolism, Guangdong Provincial Key Laboratory of New Drug Screening, School of Pharmaceutical Sciences, Southern Medical University, Guangzhou 510515, China; 3 TCM-Integrated Hospital, Southern Medical University, Guangzhou 510315, China; 4 State Key Laboratory of Natural Medicines, China Pharmaceutical University, Nanjing 210009, China

**Keywords:** magnolol, biotransformation, sulfation, liver S9 fractions, anti-inflammation

## Abstract

Magnolol, the main active ingredient of *Magnolia officinalis*, has been reported to display anti-inflammatory activity. Sulfation plays an important role in the metabolism of magnolol. The magnolol sulfated metabolite was identified by the ultra-performance liquid chromatography to quadrupole time-of-flight mass spectrometry (UPLC-Q-TOF-MS) and a proton nuclear magnetic resonance (^1^H-NMR). The magnolol sulfation activity of seven major recombinant sulfotransferases (SULTs) isoforms (SULT1A1*1, SULT1A1*2, SULT1A2, SULT1A3, SULT1B1, SULT1E1, and SULT2A1) was analyzed. The metabolic profile of magnolol was investigated in liver S9 fractions from human (HLS9), rat (RLS9), and mouse (MLS9). The anti-inflammatory effects of magnolol and its sulfated metabolite were evaluated in RAW264.7 cells stimulated by lipopolysaccharide (LPS). Magnolol was metabolized into a mono-sulfated metabolite by SULTs. Of the seven recombinant SULT isoforms examined, SULT1B1 exhibited the highest magnolol sulfation activity. In liver S9 fractions from different species, the *CL_int_* value of magnolol sulfation in HLS9 (0.96 µL/min/mg) was similar to that in RLS9 (0.99 µL/min/mg) but significantly higher than that in MLS9 (0.30 µL/min/mg). Magnolol and its sulfated metabolite both significantly downregulated the production of inflammatory mediators (IL-1β, IL-6 and TNF-α) stimulated by LPS (*p* < 0.001). These results indicated that SULT1B1 was the major enzyme responsible for the sulfation of magnolol and that the magnolol sulfated metabolite exhibited potential anti-inflammatory effects.

## 1. Introduction

Magnolol is a polyphenolic dinaphthalene compound that is isolated from the stem bark of *Magnolia officinalis* (named Houpu in Chinese) [1]. *M. officinalis* is widely used in China and other Asian countries as a traditional Chinese medicine [2]. Magnolol exhibits various pharmacological effects, including anti-inflammatory, antioxidative, anti-viral, antitumor, anti-asthma, cardiovascular protection, neuroprotection, and antibacterial activities [3,4]. Recent reports have indicated that magnolol can inhibit ulcerative colitis through the downregulation of nuclear factor-κB signaling and the upregulation of peroxisome proliferator-activated receptor-γ expression [5,6].

However, the extensive first-pass metabolism of magnolol considerably limits its oral bioavailability (which is only 4.9%) and therapeutic activity [7]. Previous studies have shown that magnolol was glucuronidated via UDP-glucuronosyltransferases (UGTs) and sulfated via sulfotransferases (SULTs) in the liver and intestine after the oral administration of magnolol in rats [7,8,9]. It was reported that UGT1A9 was the main isoform for magnolol glucuronidation [9], but it is unclear what SULT isoform is responsible for the sulfation of magnolol.

Sulfation is a significant phase II metabolic reaction mediated by SULTs, with 3′-phosphoadenosine-5′-phosphosulfate (PAPS) as a co-substrate. Many studies have been reported that sulfated metabolites, such as the sulfated metabolites of hesperidin, naringenin, and apigenin, also have pharmacological activity [10]. Similar to resveratrol, the sulfated metabolites of resveratrol also inhibited the multiplication capacity of human colorectal cancer cells and exerted an anti-inflammatory effect [11,12]. However, the bioactivity of the magnolol sulfated metabolite has not yet been determined.

There may be species differences in the metabolism of magnolol, which may affect its efficacy and toxicity. For example, when Kunming mice were administered magnolol (6.25 mg/day) for 3 months, renal injury with elevated levels of serum creatinine, urea nitrogen, and serum albumin was observed [8]. Nevertheless, when Sprague–Dawley rats were fed a diet with magnolia bark extract (containing 95.5% magnolol) for 21 days, no treatment-related microscopic lesions or any signs of toxicity were observed [1]. Therefore, the metabolism of magnolol in different species (such as humans, rats, and mouse) needs to be fully characterized in vitro.

The present study aimed to elucidate the metabolic sulfation characteristics of magnolol using different recombinant human SULTs enzymes and S9 fractions from various species in vitro. In addition, the anti-inflammation effect of a magnolol sulfated metabolite was investigated. It was reported that the Huanglian-Houpo decoction, which was the pharmaceutical product containing magnolol, was used to treat seasonal epidemic colds and influenza infections [13]. The chaiqin chengqi decoction contained the main compound of magnolol has been safely used to treat patients with acute pancreatitis [14]. Therefore, our results will provide a basis for comprehensively understanding the metabolic profile of magnolol sulfation, which will be useful for clinical pharmacists to prescribe magnolol-containing products.

## 2. Materials and Methods

### 2.1. Chemicals and Reagents

Magnolol (purity: >98%) and testosterone (used as an internal standard (IS)) were purchased from Chengdu Mansite Pharmaceutical Co. Ltd. (Chengdu, China). Pooled human liver S9 fraction mixed (HLS9) and recombinant human SULT isoforms (SULT1A1*1, 1A1*2, 1A2, 1A3, 1B1, 1E1, 2A1) were purchased from BD Biosciences (Woburn, MA, USA). Potassium phosphate dibasic (K_2_HPO_4_) and potassium dihydrogen phosphate (KH_2_PO_4_) was also obtained from BD Gentest Corp. (Woburn, MA, USA). 3′-phosphoadenosine-5′-phosphosulfate lithium salt hydrate (PAPS), magnesium chloride (MgCl_2_), and lipopolysaccharide (LPS) were purchased from Sigma-Aldrich (St. Louis, MO, USA). The RAW264.7 (murine macrophage) cell line was provided by the cell bank of the Chinese Academy of Sciences (Shanghai, China). Cell Counting Kit-8 (CCK-8) was obtained from Meilunbio (Dalian, China). All other reagents were available for analytic or highest commercially grades.

### 2.2. Animals

A male Sprague–Dawley rat (180–220 g, 10 weeks old) and C57 mouse (18–20 g, 40 days old) were purchased from Experimental Animal Center of Southern Medical University (Guangzhou, China). Separated pools of rat (*n* = 6/group) and mouse (*n* = 6/group) livers were used to prepare S9 fractions (rat liver S9 (RLS9) and mouse liver S9 (MLS9)) according to the methods previously reported [15]. All of the experiments with animals were approved by the Institution Animal Care and Use Committee of Southern Medical University (Guangzhou, China).

### 2.3. Cell Culture

RAW264.7, a monocyte-/macrophage-like cell line, is the most used cell line in in vitro studies for screening the anti-inflammatory activity of natural compounds. LPS can upregulate many inflammatory mediators such as interleukin-1beta (IL-1β), interleukin-6 (IL-6), and tumor necrosis factor alpha (TNF-α) in RAW 264.7 cells [16,17,18]. The cell line RAW264.7 was cultured in Dulbecco’s Modified Eagle’s medium high-glucose normal-culture medium containing antibiotics (100 U/mL penicillin and 100 mg/mL streptomycin) and 10% heat-inactivated fetal bovine serum at conditions of 37 °C and 5% CO_2_ in a humidified incubator.

### 2.4. Magnolol Metabolism by the Liver S9 Fractions of Different Species and Seven Recombinant Human SULTs

The sulfation reaction system and incubation procedures were the same as those in a previous study [19]. In brief, the incubation mixture, with a total volume of 200 μL, consisted of 10 μL of enzymes (liver S9 fractions), 5 μL of MgCl_2_ (1 mM), 5 μL of PAPS (0.05 mM), 178 μL of potassium phosphate buffer (KPI) (44.5 mM, pH 7.4) and 2 μL of magnolol dissolved in dimethyl sulfoxide (DMSO) (100 mM) and then diluted by 50% ice-cold acetonitrile to different concentrations. The mixture, prepared in triplicates, was incubated in a shaking water bath at 37 °C for 90 min. The reactions were stopped by the addition of an acetonitrile solution containing IS. Incubations without PAPS were used as control.

The sulfation of magnolol at different concentrations was investigated using seven recombinant human SULTs (SULT1A1*1, 1A1*2, 1A2, 1A3, 1B1, 1E1, and 2A1). All assays were conducted at 37 °C for 90 min with a final protein concentration of 0.01 mg/mL. All samples were centrifuged at 14,000× *g* for 30 min at 4 °C, and the supernatant was analyzed directly by ultra-performance liquid chromatography–tandem mass spectrometry (UPLC–MS/MS) (Agilent technologies, Palo Alto, Santa Clara, CA, USA).

### 2.5. Identification and Quantification of Magnolol and Its Sulfated Metabolite

In order to determine the molecular weight of magnolol and its sulfated metabolite, a coupled ultra-performance liquid chromatography to quadrupole time-of-flight mass spectrometry (UPLC-Q-TOF-MS) system was used to identify the metabolite. UPLC-MS/MS analysis was performed using multiple reaction monitoring (MRM) in the negative ion mode for magnolol and its sulfated metabolite using the transitions *m*/*z* 265.1 → 247.1, and *m*/*z* 345.1 → 265.1, respectively. For IS, the transition *m*/*z* 288.1 → 273.0 in the positive ion mode was used. The optimum ion source parameters were as follows: capillary voltage, 3 kV; cone voltage, 30 V; ion-source temperature, 105 °C; desolvation gas, nitrogen; and temperature, 350 °C. The purification of the magnolol sulfated metabolite was performed using a Dionex U300 HPLC System (Thermo Fisher Scientific, Waltham, MA, USA) after incubation with RSL9 for 6 h at 37 °C. To further identify the magnolol sulfated metabolite, proton magnetic resonance (^1^H-NMR) spectra were obtained with a Bruker Avance 400 spectrometer (Bruker Biospin GmbH, Rheinstetten, Germany) with deuterated dimethyl sulfoxide (DMSO-*d*_6_) as a solvent.

### 2.6. Enzyme Kinetic Studies

The incubation conditions of magnolol sulfation were optimized using different substrate (magnolol) concentrations (0, 10, 25, 50, and 80 μM), different incubation times (0, 30, 60, 90, and 120 min), and different protein concentrations (0, 0.0625, 0.125, 0.25, and 0.5 mg/mL) in the S9 fractions from different species (HLS9, RLS9, and MLS9). The reaction rate increases with increasing substrate concentration, asymptotically approaching the maximum rate *V_max_*. The concentration of a substrate should not change significantly over the course of the incubation to satisfy the steady-state assumption. Up to 10% substrate consumption during an experiment is generally considered acceptable and has minimal impact on the estimated *K_m_* and *V_max_* values. Further studies should be conducted with an appropriate incubation time and enzyme concentration, and the formation of metabolites should be under linear conditions. The enzyme kinetics of magnolol sulfation was evaluated in different S9 fractions and with different SULTs at different concentrations of magnolol according to the above conditions. The kinetic parameters were obtained by fitting the proper models such as Michaelis–Menten (V = *V_max_* × S/*K_m_* + S) and substrate inhibition (V = *V_max_* × S/[*K_m_* + S × (1 + S/*K_si_*)]) to the substrate concentrations and initial rates using GraphPad Prism 7.0 software, where *V_max_* is the maximal velocity and *K_m_* is the substrate concentration at the half-maximal rate, aided by the profiles of the Eadie Hofstee plots [20].

### 2.7. Assessment of the Anti-Inflammatory Effects of Magnolol and Its Sulfated Metabolite

The cytotoxicity of magnolol and its sulfated metabolite on RAW 264.7 cells were assessed by a CCK-8 assay. In brief, cells were treated with magnolol (0–200 μM) and magnolol sulfated metabolite (0–200 μM) for 24 h. Then, the CCK-8 solution was added into each well, followed by 4 h incubation, and optical density at 450 nm was determined. RAW264.7 cells (2 × 10^5^ cells/well) were cultured in 24-well plates with or without pretreatment with LPS (1 µg/mL) for 4 h, then incubated with magnolol or its sulfated metabolite at a series of concentrations for 24 h. The cultured RAW 264.7 cells were collected, and gene expression of related inflammatory factors (IL-1β, IL-6, and TNF-α) were analyzed by reverse transcription–quantitative PCR (RT–qPCR). PCR primers were synthesized by Tsingke Biotechnology Co., Ltd., (Beijing, China). The forward and reverse primers sequence for IL-1β were 5′-CACCTCTCAAGCAGAGCACAG-3′ and 5′- GGGTTCCATGGTGAAGTCAAC-3′, for IL-6 were 5′-CCAAGACCATCCAACTCATCTTG-3′ and 5′-TAGAGCCACCAATCCACACA-3′, for TNF-α were 5′-CCAGGTTCTCTTCAAGGGACAA-3′ and 5′- CTCCTGGTATGAAATGGCAAATC-3′, for β-actin were 5′- TGACAGGATGCAGAAGGAGA-3′ and 5′- TAGAGCCACCAATCCACACA-3′. Total RNA was extracted from RAW264.7 cells using Trizol reagent (Takara, Kyoto, Japan), according to the manufacturer’s instructions. cDNAs were reverse-transcribed from total RNA (1 µg) using a Color Reverse Transcription Kit, according to the manufacturer’s instructions (EZBioscience, Roseville, CA, USA). In brief, reverse transcription (RT) mixtures were prepared from 10 μL of the RNA sample, 5 μL of 5 × RT Master Mix and 5 μL of nuclease-free double-distilled water. The mixtures were incubated at 42 °C for 15 min, followed by 95 °C for 30 s. The product mixture was used in qPCR reactions immediately or stored at −80 °C until further analysis. qPCR analyses were performed in a LightCycler 480 II instrument (Roche Molecular Systems, Inc., Forrenstrasse, Rotkreuz, Basel, Switzerland) using a SYBR green/Rox qPCR master mix. The thermocycle program consisted of three steps: an initial denaturation step at 95 °C for 30 s, followed by 45 cycles at 95 °C for 10 s, 60 °C for 30 s, and 72 °C for 30 s, and a melt curve stage at 95 °C for 1 min, 55 °C for 1 min, 95 °C for 10 s, and 40 °C for 1 min. Data were analyzed according to the 2^–ΔΔCT^ method [21], and β-actin was used as an endogenous control.

### 2.8. Data Analysis

For variance with or without Tukey–Kramer multiple comparison tests, one-way ANOVA analysis was used to evaluate statistical differences in SPSS Statistics (Version 17.0, SPSS Inc., Chicago, IL, USA). For normally distributed data, Pearson’s product–moment correlations were performed to assess correlation analyses. Differences were considered significant when *p*-values were less than 0.05.

## 3. Results

### 3.1. Identification of Magnolol Sulfated Metabolite by UPLC-Q-TOF-MS and ^1^H-NMR

**Figure 1:**
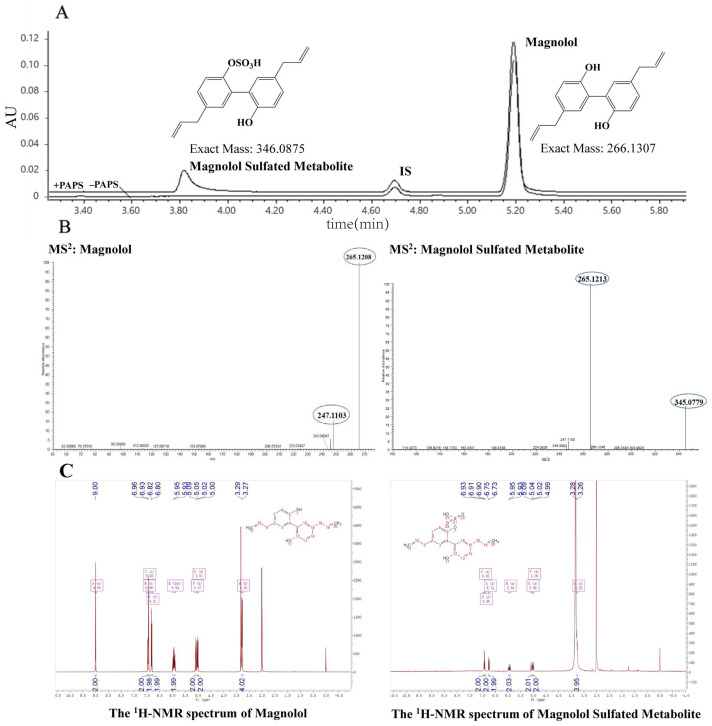
(**A**) Representative ultraperformance liquid chromatography chromatogram for the quantitative analyses of magnolol and its sulfated metabolite in HLS9. (**B**) Representative MS/MS (MS^2^) spectra of magnolol and its sulfated metabolite. (**C**) Representative ^1^H-NMR spectra of magnolol and its sulfated metabolite.

A magnolol sulfated metabolite was observed after incubation with liver S9 fractions in the presence of PAPS (Figure 1A). This finding indicated that magnolol was metabolized by SULTs. The mass spectrum of magnolol and its sulfated metabolite were dominated by the ion at *m*/*z* 265.1213 [M–H]^−^ and *m*/*z* 345.0786 [M–H]^−^ respectively (Figure 1B). The difference of 79.9573 Da (SO_3_) between magnolol and its sulfated metabolite indicated that mono-sulfated magnolol was formed. Compared with the ^1^H-NMR spectrum of magnolol, the H-7 singlet at 9.00 ppm had disappeared in the magnolol sulfated metabolite characteristically as a singlet and the H-6 proton a had minor shift, while the other protons had no chemical shifts (Figure 1C, Table 1).

### 3.2. Kinetic Analysis of Magnolol Sulfation in Liver S9 Fractions from Different Species

**Figure 2:**
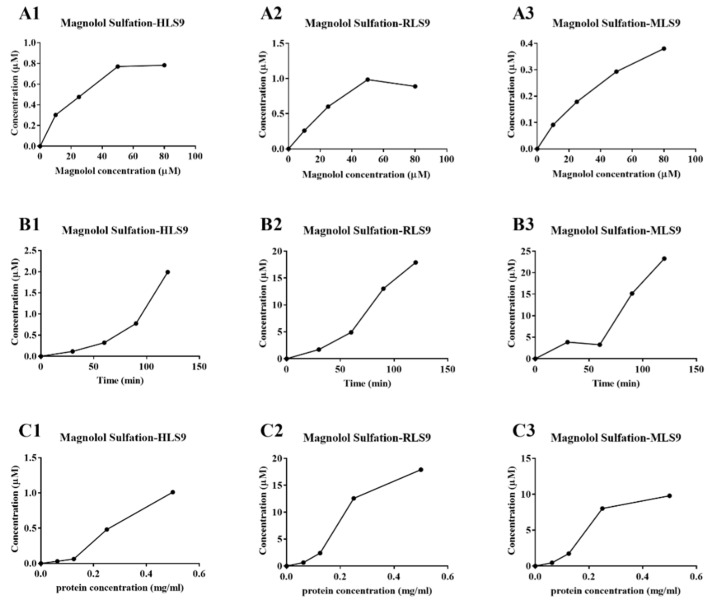
Substrate concentration (**A1**–**A3**), incubation time (**B1**–**B3**), and protein concentration (**C1**–**C3**)—dependent incubation studies of magnolol sulfation metabolism in the liver S9 fractions from different species (HLS9, RLS9, and MLS9). Each point represents an average of three determinations, and the error bar is the standard deviation of the mean.

As shown in Figure 2(A1)–(A3), when the concentration of magnolol was 25 μM, the substrate consumption was approximately 10% in S9 fractions from different species (HLS9, RLS9, and MLS9), which had minimal impact on estimated *K_m_* and *V_max_* values. As shown in Figure 2(B1)–(B3), an incubation time of 90 min for all of the liver S9 fractions from different species (HLS9, RLS9, and MLS9) was suitable for conducting further experiments. As shown in Figure 2(C1)–(C3), a maximum rate of metabolite formation was observed for all of the S9 fractions (HLS9, RLS9, and MLS9) at 0.25 mg protein/mL of enzyme. A magnolol concentration of 25 μM, incubation time of 90 min, and protein concentration of 0.25 mg protein/mL were determined to be the optimum conditions for magnolol sulfation.

**Figure 3:**
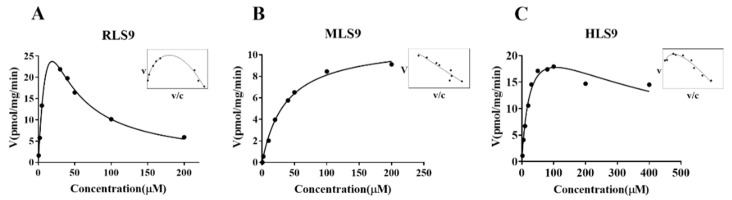
Enzyme kinetic analysis for the magnolol sulfation by RLS9 (**A**), MLS9 (**B**) and HLS9 (**C**). In each figure, the inset shows the Eade–Hofstee plot. Each point is the average of three determinations, and the error bar is the standard deviation of the mean.

To characterize the SULT enzymatic activities of the different species (RLS9, MLS9, and HLS9), enzymatic assays were performed using different concentrations of magnolol. The sulfation reaction of magnolol in RLS9 and HLS9 was best-fitted by a substrate inhibition equation (Figure 3A,C), whereas in MLS9, it was best-fitted by a Michaelis–Menten equation (Figure 3B). The enzyme kinetic parameters, *K_m_*, *V_max_*, and *CL_int_* values, are shown in Table 2. The sulfation of magnolol in HLS9 and RLS9 showed a markedly higher *V_max_* value (31.15 ± 2.966 and 26.82 ± 3.214 pmol/mg/min, respectively) along with a lower *K_m_* value (26.85 ± 6.797 μM, and 32.25 ± 8.166 μM, respectively) than in MLS9 (*V_max_*: 11.12 ± 0.3647 pmol/mg/min, *K_m_*: 36.82 ± 3.365 μM).

The *CL_int_* value of magnolol sulfation in HLS9 (0.96 µL/min/mg) was similar to that in RLS9 (0.99 µL/min/mg) but higher than that in MLS9 (0.30 µL/min/mg).

### 3.3. Magnolol Sulfation by Seven Recombinant Human SULT Isoforms

**Figure 4:**
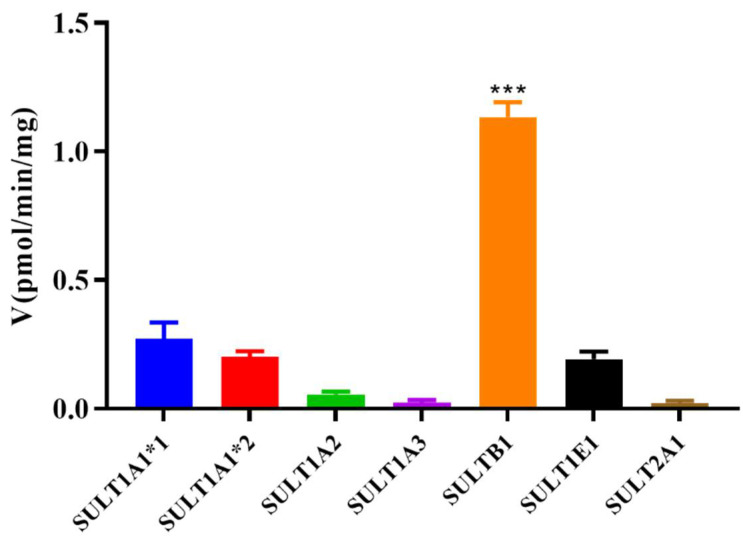
Identification of the major SULT enzymes responsible for the sulfation of magnolol. Formation rate of magnolol sulfated metabolite in the incubation of magnolol (25 μM) with seven recombinant human SULT isoforms (0.05 mg protein/mL). It was calculated and expressed as a nanomole per minute per milligram of protein for sulfation rates. *** *p* < 0.001 vs other group. Each point is the average of three determinations, and the error bar is the standard deviation of the mean.

**Figure 5:**
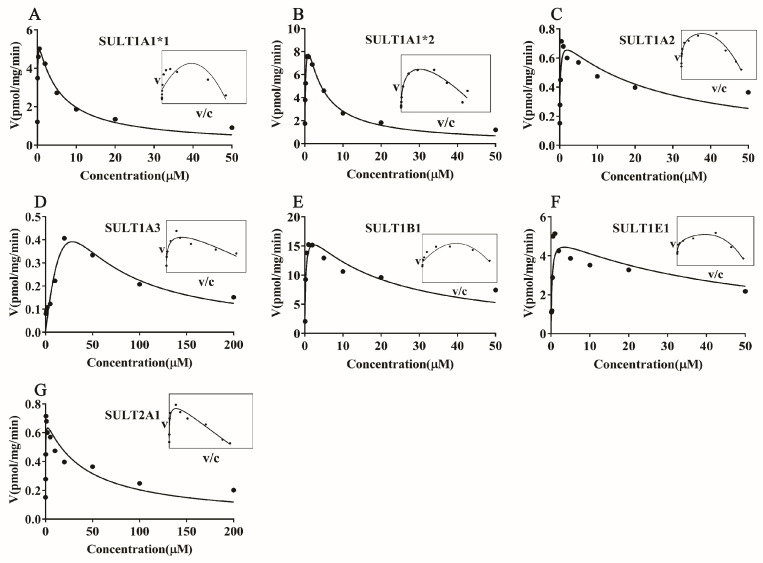
Enzyme kinetic analysis for the activities of the seven recombinants human SULT isoforms (**A**–**G**) through the magnolol substrate. Results obtained were analyzed using GraphPad Prism 7.0 software. In each figure, the inset shows the Eade–Hofstee plot. Each point is the average of three determinations, and the error bar is the standard deviation of the mean.

To determine the main SULT isoform involved in the sulfation of magnolol, experiments were carried out with seven recombinant human SULT isoforms (SULT1A1*1, SULT1A1*2, SULT1A2, SULT1A3, SULT1B1, SULT1E1, and SULT2A1). The results suggested that all seven recombinant human SULT isoforms, especially SULT1B1, showed metabolic activity toward magnolol (*p* < 0.001) (Figure 4). Further kinetics studies of magnolol sulfation were performed using seven recombinant human SULT isoforms. The results indicated that the variation of the rate with the substrate (magnolol) concentration fitted the substrate inhibitor equation for all samples. SULT1B1 exhibited the highest magnolol sulfation activity, with a *CL_int_* value of 65.27 μL/min/mg, followed by SULT1A1*2 (40.75 μL/min/mg) > SULT1E1 (19.65 μL/min/mg) > SULT1A1*1 (12.12 μL/min/mg) > SULT2A1 (5.07 μL/min/mg) > SULT1A2 (4.75 μL/min/mg) > SULT1A3 (0.03 μL/min/mg) (Figure 5, Table 3).

### 3.4. Correlation Study

**Figure 6:**
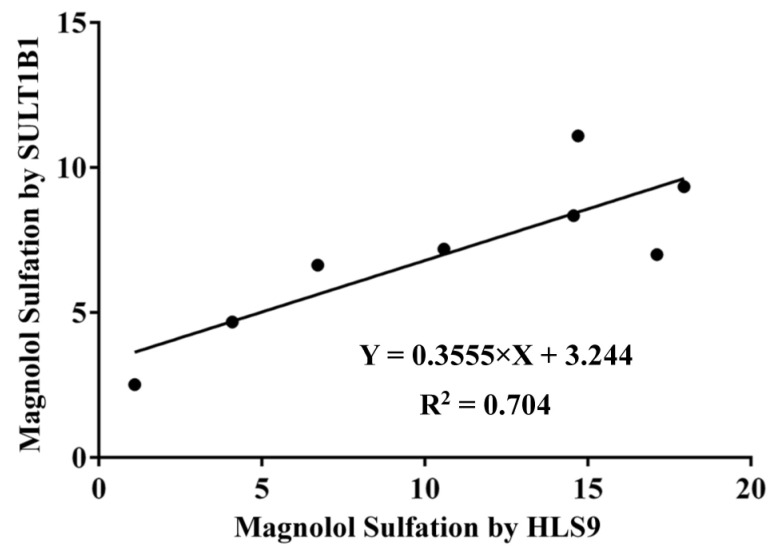
Correlation analysis between the sulfation activities of human liver S9 (HLS9) and SULT1B1. Correlation analysis were performed using Pearson’s product–moment correlation for normally distributed data.

Linear regression was used to derive apparent correlations between the sulfation activities of SULT1B1 and HLS9 for the same series of substrate concentrations used in the kinetic studies of magnolol. Strong correlations were observed between the magnolol sulfation metabolism in HLS9 and magnolol sulfation metabolism by the SULT1B1 isoform (R^2^ = 0.704, Figure 6) [22,23]. This correlation suggested that SULT1B1 may be the major SULT isoform responsible for the sulfation of magnolol in the human liver S9 fraction in vivo.

### 3.5. Effect of Magnolol and Magnolol Sulfated Metabolite on the Expression of IL-1β, IL-6, and TNF-α in LPS-Stimulated RAW264.7 Cells

**Figure 7:**
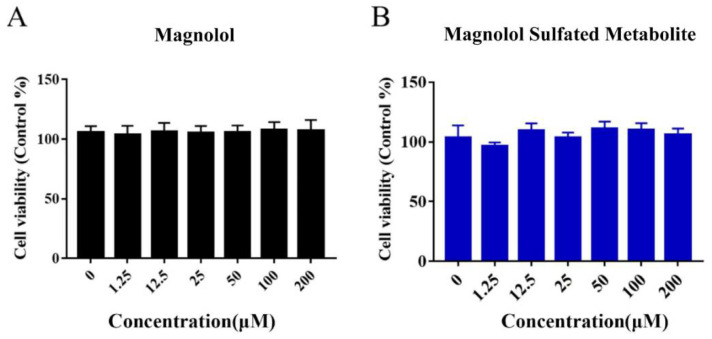
The effect of magnolol (**A**) and its sulfated metabolite (**B**) on RAW264.7 cell viability at a range concentration.

**Figure 8:**
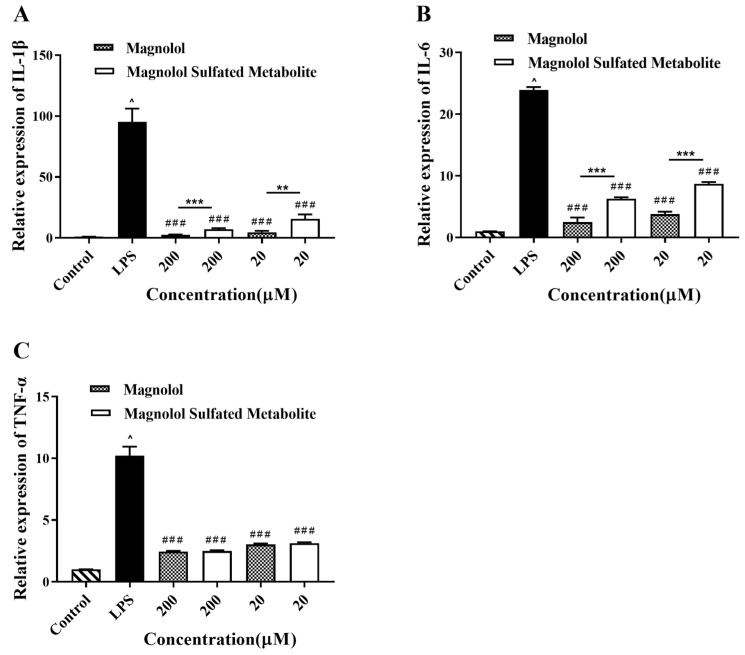
The effect of magnolol and its sulfated metabolite on the expression of IL-1β (**A**), IL-6 (**B**), and TNF-α (**C**) in LPS- stimulated RAW264.7 cells. Each data represents an average of four determinations, and the error bar is the standard deviation of the mean. ^^^ *p* < 0.05 vs. control. ^###^ *p* < 0.001 vs. LPS group. ** *p* < 0.01, *** *p* < 0.001 vs. the magnolol group.

As shown in Figure 7, no effect on cell viability was observed over a concentration range of 1.25–200 μM magnolol or magnolol sulfated metabolite. As shown in Figure 8, IL-1β, IL-6, and TNF-α expression levels were significantly increased by LPS simulation. Magnolol and its sulfated metabolite both significantly reduced the mRNA levels of inflammatory mediators (IL-1β, IL-6, and TNF-α) stimulated by LPS (*p* < 0.001). The inhibited effects on the expression of IL-1β and IL-6 by magnolol were significantly greater than that of magnolol sulfated metabolite (*p <* 0.001), but no difference was observed on the expression of TNF-α. These results suggested that magnolol sulfated metabolite has also exhibited significant anti-inflammatory activity.

## 4. Discussion

We first fully elucidated the profiles of magnolol metabolized by SULTs. Previous studies have reported that magnolol and honokiol were hydroxylated bisphenol isomers [7,24,25]; thus, the structural differences induce the observed differences in oral bioavailability [26]. Although the oral absolute bioavailability of magnolol (4.9%) has been shown to be higher than that of honokiol (3.6%), magnolol still has a low bioavailability in rats after oral administration [7,27,28]. A previous study has shown that magnolol contains two hydroxyl groups at ortho-positions, which form an intramolecular hydrogen bond easily and may influence the metabolic process of magnolol [29]. The poor bioavailability of magnolol might be partly because of its metabolism through glucuronidation, and sulfation is the predominant metabolite in rats [24]. A previous study has shown that UGT1 was involved in the glucuronidation of magnolol in human liver microsomes [9]. However, the structures of magnolol sulfated metabolite and the SULT isoforms responsible for magnolol metabolism have not been comprehensively determined. Therefore, this study was undertaken to identify the magnolol sulfated metabolite in liver S9 fractions using UPLC-Q-TOF-MS and ^1^H-NMR, and the results indicated that mono-sulfated, not disulfate magnolol, was generated in liver S9 fractions in vitro in experiments with different substrate concentrations, times, and protein concentrations (Figure 1). Animal models have been commonly used in preclinical studies to investigate pharmacokinetics, bioactivity, and toxicity in humans [30]. A suitable animal model used for preclinical and basic studies should have metabolic patterns similar to those in humans, including identical or similar metabolic activities, enzymes, and catalytic processes [31]. Herein, a comparative study of magnolol sulfation in human and two common experimental animal species (rat and mouse) was performed to determine the catalytic efficiency of sulfation in liver S9 fractions. The results indicated that the rat, but not the mouse, could be a suitable model for extrapolating the sulfation metabolism of magnolol to humans (Figure 3).

The SULT enzymes responsible for the formation of magnolol sulfation were identified using seven recombinant human SULT enzymes. It has been reported that SULT1A1*1, SULT1A1*2, SULT1A2, SULT1A3, SULT1B1, SULT1E1, and SULT2A1 are the most important human liver drug-metabolizing SULT isoforms [32]. Enzymes in the SULT1 family (especially SULT1B1, SULT1A1*1, and SULT1A1*2) showed higher metabolic activity than the SULT2A1 isoform toward magnolol (Figure 4). SULT1A1 and SULT1B1 make up almost 70% of the SULTs family present in the liver; thus, they are generally considered the major enzymes in humans [32,33]. Most importantly, members of the SULT1 (phenol sulfotransferase) family have been shown to preferentially sulfate phenolic compounds [34]. Kinetics studies of magnolol sulfation were performed using seven recombinant human SULT isoforms. The results indicated that SULT1B1 exhibited the highest magnolol sulfation activity, followed by SULT1A1*2 > SULT1E1 > SULT1A1*1 > SULT2A1 > SULT1A2 > SULT1A3 (Figure 5, Table 3). Previous studies have indicated that phenol and catechol compounds had inhibitory effects on the SULT1A family [35]. Similarly, in the present study, magnolol sulfation mediated by the SULTs was consistent with the substrate inhibition equation (Figure 5). The inhibition of SULT activity by magnolol may increase the risk of toxicity and the potential for drug–drug interactions. The mechanism of substrate inhibition may be explained by a hypothetical two-site model in which one binding site is productive, and the other site is inhibitory and operable at high substrate concentrations [36]. However, another explanation is probably more reasonable for the substrate inhibition of magnolol. In this mechanism, the binding of the aglycone substrate to the enzyme–SULT complex leads to a nonproductive dead-end complex, which could slow the completion of the catalytic cycle [37]. Future studies should focus on determining the potency of the magnolol inhibition of different SULT isoforms. Additionally, high correlation was observed between the sulfation rate of magnolol with SULT1B1 and the rate in HLS9 (Figure 6). When combined with other drugs that are primarily metabolized by SULT1B1, such as oxymorphone, nalbuphine, nalorphine, naltrexone, and some steroid hormones [38], the metabolism and pharmacokinetics of endogenous and exogenous magnolol may be altered because of the metabolic interaction mediated by SULT1B1 [39]. Thus, attention should be paid to when other drugs are combined with magnolol.

The metabolism of a drug can have an effect on its pharmacological activity. In general, the conjugated metabolites of Phase II metabolism have increased molecular weight and become less active than the parent drug. It has been reported that the sulfation metabolite of melatonin exhibited as potent bioactivity as melatonin [40]. The sulfation metabolites of resveratrol also exert a strong anti-inflammatory effect [11,12]. The effect of sulfation on the anti- inflammatory activity of magnolol was explored in the present study. We found that magnolol and its sulfated metabolite both significantly downregulated the production of the inflammatory mediators (IL-1β, IL-6, and TNF-α) stimulated by LPS (Figure 8).

It has been reported that *M. obovata* extracts (the main ingredient was magnolol) were used as a plant-derived natural preservative in cosmetic products [41]. Furthermore, magnolol has relatively low cytotoxic effects, suggesting the possibility of introducing them as safe topical therapeutic agents for acne [42]. As we know, the skin contains a huge number of metabolizing enzymes that promote the biotransformation reaction for many compounds. For example, vitamin D_3_ and lumisterol (L_3_) were transformed to active metabolites through the skin metabolizing enzymes, which exerted a variety of antiaging effects [43]. Hence, the cosmetic products containing magnolol may be metabolized when applied on the skin. According to our studies, magnolol sulfated metabolite had a great anti-inflammatory effect. We speculated that the pharmacodynamics of cosmetic products containing magnolol cannot be affected on the skin. Therefore, we will investigate the metabolic pathways of cosmetic products containing magnolol when applied on the skin in the future.

## 5. Conclusions

Our present study filled a gap in the understanding of the metabolism of magnolol through sulfation. The rat but not the mouse could be a suitable model for extrapolating the sulfation metabolism of magnolol to humans. SULT1B1 was the major enzyme responsible for the sulfation of magnolol, and the magnolol sulfated metabolite exhibited a potential anti-inflammatory effect.

## Figures and Tables

**Table 1:** ^1^H-NMR chemical shifts (δ) of magnolol and magnolol sulfated metabolite.

Proton	Magnolol (ppm)	Magnolol Sulfated Metabolite (ppm)
7	9.0	-
11	5.00	4.99
10	5.91	5.90
9	3.27	3.27
1	6.90	6.93
6	6.74	6.81

**Table 2:** Kinetic parameters derived for magnolol sulfation in liver S9 fractions from different species. Data represents the mean ± standard deviation of three determinations. SI stands for the substrate inhibition model.

Species	*K_m_* (μM)	*V_max_* (pmol/mg/min)	*CL_int_* (*V_max_*/*K_m_* μL/min/mg)	Kinetic Mechanism Model
RLS9	32.25 ± 8.166	31.15 ± 2.967	0.96	SI
MLS9	36.82 ± 3.365	11.12 ± 0.365	0.30	MM
HLS9	26.85 ± 6.797	26.82 ± 3.214	0.99	SI

**Table 3:** Summarized enzyme kinetic parameters of seven human recombinant SULT isoforms with magnolol as the substrate. SI stands for the substrate inhibition model. Data represents the mean ± standard deviation derived from three determinations.

Isoform	*K_m_* (μM)	*V_max_* (pmol/mg/min)	*CL_int_* (*V_max_*/*K_m_* μL/min/mg)	Kinetic Mechanism Model
SULT1A1*1	0.49 ± 0.01	5.94 ± 0.32	12.12	SI
SULT1A1*2	0.32 ± 0.06	13.04 ± 1.24	40.75	SI
SULT1A2	0.16 ± 0.07	0.76 ± 0.09	4.75	SI
SULT1A3	69.34 ± 149.80	2.29 ± 4.31	0.03	SI
SULT1B1	0.29 ± 0.15	18.93 ± 3.16	65.27	SI
SULT1E1	0.26 ± 0.16	5.11 ± 0.90	19.65	SI
SULT2A1	0.14 ± 0.06	0.71 ± 0.07	5.07	SI

## Data Availability

T The data presented in this study are available in article.
